# The differences of fibrinogen levels in various types of hemorrhagic transformations

**DOI:** 10.3389/fneur.2024.1364875

**Published:** 2024-07-25

**Authors:** Jingfang Long, Jiahao Chen, Guiqian Huang, Zhen Chen, Heyu Zhang, Ye Zhang, Qi Duan, Beilan Wu, Jincai He

**Affiliations:** ^1^Department of Neurology, The First Affiliated Hospital of Wenzhou Medical University, Wenzhou, Zhejiang, China; ^2^Department of Neurology, Wenzhou Central Hospital, Wenzhou, Zhejiang, China; ^3^School of Mental Health, Wenzhou Medical University, Wenzhou, Zhejiang, China; ^4^Zhejiang Provincial Key Laboratory of Aging and Neurological Disorder Research, The First Affiliated Hospital of Wenzhou Medical University, Wenzhou, Zhejiang, China

**Keywords:** hemorrhagic transformation, acute ischemic stroke, fibrinogen, mechanical thrombectomy, stroke

## Abstract

**Introduction:**

Hemorrhagic transformation (HT) is a serious complication that can occur spontaneously after an acute ischemic stroke (AIS) or after a thrombolytic/mechanical thrombectomy. Our study aims to explore the potential correlations between fibrinogen levels and the occurrence of spontaneous HT (sHT) and HT after mechanical thrombectomy (tHT).

**Methods:**

A total of 423 consecutive AIS patients diagnosed HT who did not undergone thrombolysis and 423 age- and sex-matched patients without HT (non-HT) were enrolled. Fibrinogen levels were measured within 24 h of admission after stroke. The cohorts were trisected according to fibrinogen levels. The HT were further categorized into hemorrhagic infarction (HI) or parenchymal hematoma (PH) based on their imaging characteristics.

**Results:**

In sHT cohort, fibrinogen levels were higher in HT patients than non-HT patients (*p* < 0.001 versus *p* = 0.002). High fibrinogen levels were associated with the severity of HT. HT patients without atrial fibrillation (AF) had higher levels of fibrinogen compared to non-HT (median 3.805 vs. 3.160, *p* < 0.001). This relationship did not differ among AF patients. In tHT cohort, fibrinogen levels were lower in HT patients than non-HT patients (*p* = 0.002). Lower fibrinogen levels were associated with the severity of HT (*p* = 0.004). The highest trisection of fibrinogen both in two cohorts were associated with HT [sHT cohort: OR = 2.515 (1.339–4.725), *p* = 0.016; that cohort: OR = 0.238 (0.108–0.523), *p* = 0.003].

**Conclusion:**

Our study suggests that lower fibrinogen level in sHT without AF and higher fibrinogen level in tHT are associated with more severe HT.

## 1 Introduction

Hemorrhagic transformation (HT) is known as a natural progression of acute ischemic stroke (AIS) caused by the restoration of blood flow in the ischemic area (1). This condition can often lead to a deterioration of neurological function. HT includes spontaneous hemorrhage (spontaneous hemorrhagic transformation) and post-interventional hemorrhage (including thrombolysis, thrombectomy, and anticoagulation, etc.) after AIS (2). Existing findings suggest that the Asian population is significantly more prone to HT than the western population (3, 4). A study from China in 2016 reported that the incidence of HT after endovascular therapy was as high as 49.5%, with symptomatic intracranial hemorrhage (sICH) at 16.0% (5). Existing studies on HT have mainly focused on thrombolytic therapy, with fewer studies on spontaneous HT (sHT) and HT after mechanical thrombectomy (tHT).

The European Cooperative Acute Stroke Study (ECASS) classifies hemorrhagic stroke based on radiological findings, as hemorrhagic infarction (HI) and parenchymal hematoma (PH) (6). PH has been associated with a higher risk of clinical complications, as well as a longer hospital stay and a severe clinical prognosis when compared to HI, often results in death at discharge (7, 8).

The AIS is often associated with atherosclerosis, and plaque instability can lead to the formation of blood clots, resulting in abnormal cerebral blood flow (9). During the treatment window period for stroke, reperfusion therapy is an effective means of improving stroke prognosis, but there is a risk of severe hemorrhage (10). Studies have found that blood biomarkers (blood glucose, magnesium), inflammation levels, blood–brain barrier disruption, fibrinolysis/antifibrinolytic disorders, oxidative stress, etc., are associated with hemorrhagic transformation after AIS (11, 12). Several previous studies indicate that HT is connected with abnormalities in the coagulation and fibrinolysis systems (13). Fibrinogen is a clotting protein involved in primary and secondary hemostasis that plays a crucial role in platelet aggregation and building the fibrin network (13). Additionally, fibrinogen is an acute-phase protein that undergoes significant upregulation during systemic inflammation (14). Studies have reported a close relationship between fibrinogen levels and stroke prognosis. It has been reported that a good prognosis in patients with spontaneous brain hemorrhage during non-surgical treatment is associated with low levels of fibrinogen (15, 16). Hemorrhagic transformation is one of the serious complications of intravenous thrombolysis with tissue plasminogen activator (t-PA), and the vast majority of currently published studies have focused on reducing or preventing hemorrhagic transformation after thrombolysis (17, 18), and our team previously reported higher fibrinogen in HT patients who did not receive thrombolysis (19), However, there is currently limited research comparing fibrinogen with different types of HT (spontaneous hemorrhagic transformation and hemorrhagic transformation after mechanical thrombectomy).

The aim of our study is to investigate whether serum fibrinogen, an early serum biomarker, can aid in identifying stroke patients who might be at risk of HT and are appropriate for early thrombectomy, thereby enhancing clinical outcomes and guiding appropriate treatment.

## 2 Materials and methods

### 2.1 Patients

This retrospective cohort study was conducted at the Stroke Center of the First Affiliated Hospital of Wenzhou Medical University, China, and included consecutive patients aged 18 years or older confirmed the diagnosis of HT following AIS between January 2012 and February 2023.

This study was approved by the Institutional Review Board and Ethics Committee of the First Affiliated Hospital of Wenzhou Medical University. Informed consent was waived since all data were anonymized and this was a retrospective study.

Upon admission, the diagnosis of first-ever AIS was confirmed using computed tomography (CT) or magnetic resonance imaging (MRI). The exclusion criteria were: (1) patients with hemorrhagic stroke or transient ischemic attacks (TIA); (2) patients with severe liver or kidney dysfunction; (3) patients failed to receive a repeat CT/MRI scan; (4) patients received intravenous thrombolytic therapy; (5) patients with incomplete medical data.

Finally, this study included 423 consecutive patients who were diagnosed with HT after AIS, comprising of 262 patients with spontaneously occurring HT (sHT) and 161 patients with HT following thrombectomy (tHT). For each cohort, an equal number of age- and sex-matched AIS patients without HT (Non-HT) were randomly selected as controls from the Stroke Center of our institution between January 2017 and February 2023. All patients met the inclusion criteria.

### 2.2 Data collection and group stratification

Demographic characteristics, including age and sex, were collected and data including the history of atrial fibrillation (AF), diabetes mellitus, hypertension, coronary heart disease (CHD), current cigarette smoking, and current drinking status were obtained to assess stroke risk.

Laboratory tests were conducted within 24 h of hospital admission under fasting conditions. Laboratory inspection, including erythrocyte count, leukocyte count, platelet (PLT) count, hemoglobin count, fasting blood glucose, prothrombin time (PT), activated partial thromboplastin time (APTT), thrombin time (TT) and fibrinogen. Trial of ORG 10172 in Acute Stroke Treatment (TOAST) criteria were used to classify the ischemic stroke subtypes (20). In addition, the use of anticoagulant and antiplatelet therapies for AIS during hospitalization before the onset of HT was recorded. The severity of stroke was evaluated within 24 h of admission by qualified neurologists using the National Institutes of Health Stroke Scale (NIHSS) score.

To maintain adequate statistical power within each category, all patients were divided into trisection based on the distribution of their baseline serum fibrinogen levels (21). This allowed for the examination of potential improvements in performance across the trisection.

### 2.3 Diagnosis and classification of HT subtypes

Within 24 h of stroke onset, all patients underwent a brain CT scan or MRI, which included diffusion-weighted imaging (DWI) and T2-weighted gradient-echo. A subsequent CT/MRI was performed 7 ± 2 days after stroke onset or whenever the patient’s clinical condition worsened, in order to diagnose HT. Two neurologists, who were blinded to the clinical and laboratory measurements, independently evaluated the CT/MRI scans and diagnosed HT. HT was categorized radiologically according to the recommendations of the ECASS (22, 23): HI type 1 (small petechiae along the periphery of the infarct), HI type 2 (more confluent petechiae around the infarcted area without a space-occupying effect), PH type 1 (hematoma <30% of the infarcted area with a mild space-occupying effect), and PH type 2 (hematoma >30% of the infarcted area with a significant space-occupying effect).

### 2.4 Statistical analysis

The normality of the data distribution was tested using the Kolmogorov–Smirnov test. For continuous variables with normal distributions, the mean ± standard deviation was used, and for those with non-normal distributions, the median with interquartile range was used. Categorical variables were expressed as relative frequency and percentage. Student’s *t*-test or the Mann–Whitney *U* test was used to compare continuous variables, as appropriate. The chi-square test or Fisher’s exact test was used for categorical variables. One-way analysis of variance (ANOVA) or Kruskal–Wallis test was used to perform statistical comparisons of fibrinogen stratification for continuous variables, while Pearson’s chi-square test or Fisher’s exact test was used for categorical variables. To determine whether the fibrinogen stratification was an independent predictor of HT after AIS, a multivariate-adjusted conditional binary logistic regression was performed after adjusting for conventional confounding factors and significant variables (*p* < 0.1) identified in univariate conditional logistic regression analysis. A two-tailed *p* < 0.05 was taken to indicate statistical significance.

## 3 Results

**Figure 1:**
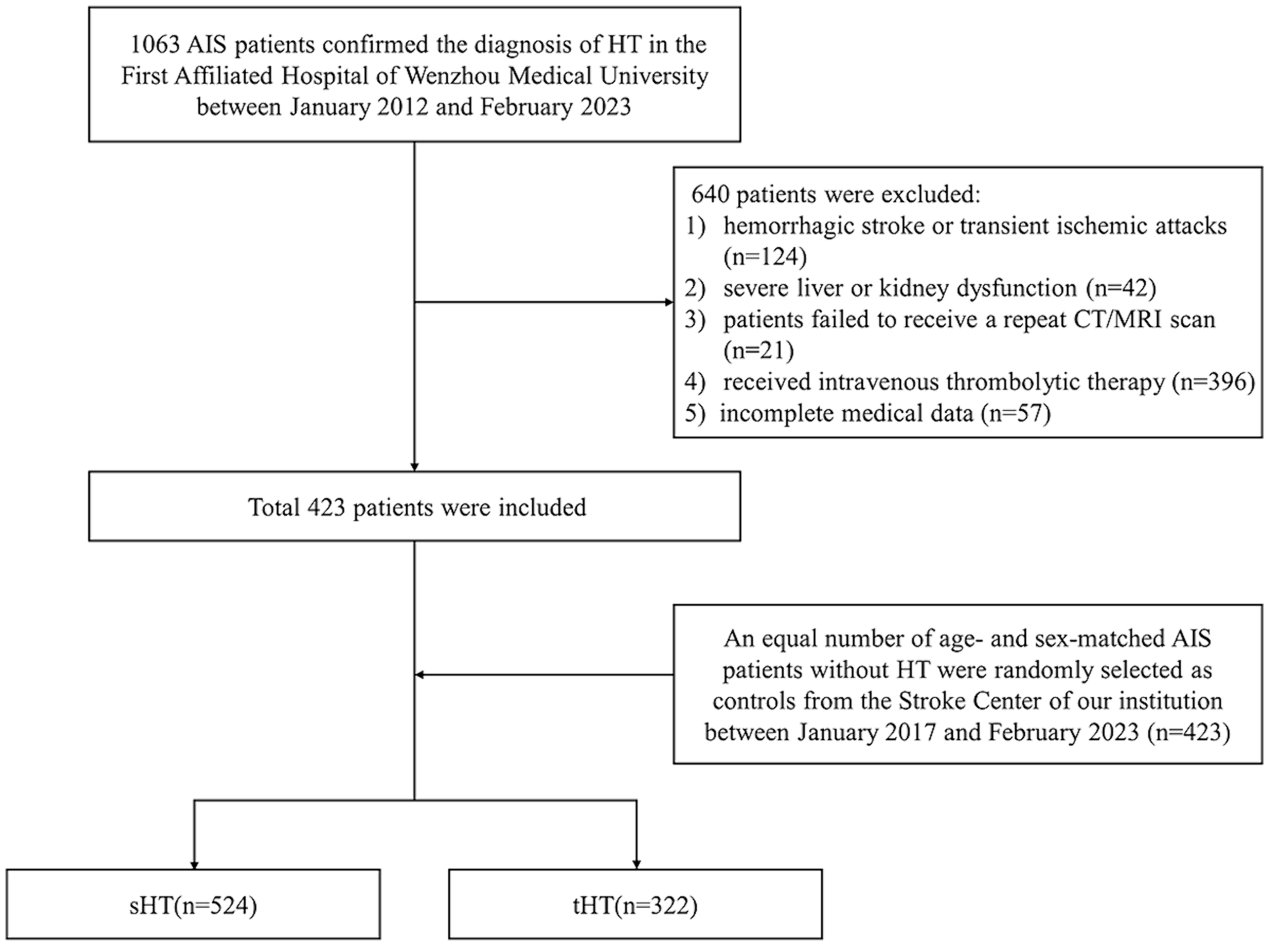
Study flow diagram.

This study enrolled 846 stroke patients divided in two cohorts, including 322 with MT treatment and 524 without MT treatment (Figure 1).

### 3.1 Baseline characteristics of patients in the two cohorts

Supplementary Table S1 shows the baseline characteristics and laboratory findings of AIS patients. The median age of the sHT cohort is 67(interquartile range, 59–74), with 373 males (71.2%), and the NIHSS score at discharge is 5 (1–10). The median age of the tHT cohort is even older, at 70 (interquartile range, 61–77, *p* < 0.001), with 222 males (68.9%), and a NIHSS score of 10 (4–19) at discharge. The tHT cohort has a higher history of AF, with 127 cases (39.4%), and a higher history of hyperlipidemia, with 187 cases (58.1%). The fibrinogen levels in the tHT cohort were lower than those in the sHT cohort (*p* = 0.002). The differences in the baseline characteristics of the AIS patients with and without HT in the two cohorts are shown in Table 1, patient with HT have higher mRS and NIHSS scores on both admission and discharge, higher RBC, WBC, CRP level than patients without HT. In sHT cohort, the level of fibrinogen of HT patients is higher than non-HT patients (3.76 versus 3.21 g/L, *p* < 0.001), while in tHT cohort, fibrinogen level in patients with HT is lower than non-HT patients (2.91 versus 3.10 g/L, *p* = 0.002). HT patients are more likely to receive anticoagulants and antiplatelets treatment in sHT cohort.

**Table 1:** Differences in the baseline characteristics of the AIS patients with and without HT in the two cohorts.

	sHT cohort			tHT cohort		
Variables	Non-HT (*n* = 262)	HT (*n* = 262)	*p*-value*	Non-HT (*n* = 161)	HT (*n* = 161)	*p*-value*
Demographic parameters						
Age (years)	68(59–73)	66(58–75)	0.957	70(62–76)	70(61–78)	0.921
Sex (male, *n*%)	186(71.0%)	187(71.3%)	1.000	112(60.6%)	110(68.3%)	1.000
Vascular risk factors						
TOAST *n* (%)			0.207			0.184
Large artery	223(85.1%)	220(84.0%)		80(49.7%)	69(42.9%)	
Cardioembolism	26(9.9%)	35(13.4%)		61(37.9%)	77(47.8%)	
Others	13(5.0%)	7(26.7%)		20(12.4)	15(9.3%)	
History of AF, *n* (%)	26(9.9%)	55(21.0%)	**<0.001**	60(37.3%)	67(41.6%)	0.494
History of hypertension, *n* (%)	162(61.8%)	160(61.1%)	0.929	111(68.9%)	109(67.7%)	0.905
History of diabetes, *n* (%)	72(27.5%)	70(26.7%)	0.922	32(19.9%)	37(23.0%)	0.587
History of hyperlipemia	19(7.3%)	20(7.6%)	1.000	97(60.2%)	90(55.9%)	0.532
History of coronary heart disease, *n* (%)	23(8.8%)	23(8.8%)	1.000	19(11.8%)	21(13.0%)	0.866
History of stroke	20(7.6%)	29(11.1%)	0.239	27(17.8%)	16(9.9%)	0.101
Current smoking, *n* (%)	89(34.0%)	88(33.6%)	1.000	65(40.4%)	58(36.0%)	0.491
Current drinking, *n* (%)	73(27.9%)	79(30.2%)	0.630	53(32.9%)	54(33.5%)	1.000
mRS on admission, median (IQR)	2(1–3)	3(2–4)	**<0.001**	4(4–5)	5(4–5)	**0.033**
mRS on discharge, median (IQR)	2(1–3)	2(1–3)	**<0.001**	4(2–5)	4(4–5)	**<0.001**
NIHSS on admission, median (IQR)	4(2–8)	9(5–13)	**<0.001**	15(10–20)	16(12–24)	0.093
NIHSS on discharge, median (IQR)	3(1–10)	7(3–11)	**<0.001**	8(2–15)	14(7–24)	**<0.001**
Biochemistry and vital signs on admission						
RBC (×10^12^/L)	4.5(4.2–4.8)	4.4(4.1–4.8)	0.224	4.00 ± 0.63	4.12 ± 0.61	0.097
WBC (×10^9^/L)	7.2(5.9–8.4)	7.5(6.1–9.5)	**0.031**	9.0(7.1–11.1)	10.1(8.4–12.4)	**<0.001**
Hb (g/L)	139.0(128.0–147.0)	136.0(126.0–145.0)	0.129	124.0(109.0–135.0)	127.0(113.0–140.0)	**0.027**
PLT (×10^9^/L)	198.0(173.0–230)	186.0(150.0–230.0)	**0.002**	191.0(155.0–243.0)	178.0(144.0–219.0)	**0.049**
Glucose (mmol/L)	5.0(4.5–6.5)	4.5(4.9–7.2)	**<0.001**	6.4(5.5–8.0)	7.7(6.3–10.0)	**<0.001**
ALT (U/L)	19.0(14.0–27.0)	19.0(13.0–29.0)	0.772	16.0(11.0–23.0)	19.0(13.0–29.0)	**0.006**
AST (U/L)	22.0(19.0–30.0)	26.0(20.0–33.0)	**<0.001**	22.0(19.0–28.0)	24.0(20–34)	**0.027**
Fibrinogen (g/L)	3.21(2.79–3.9)	3.76(3.05–4.72)	**<0.001**	3.10(2.65–3.78)	2.91(2.49–3.31)	**0.002**
PT (s)	13.3(12.8–13.8)	13.8(13.2–14.4)	**<0.001**	14.0(13.4–14.6)	14.2(13.3–15.0)	0.212
APTT (s)	36.0(34.2–38.3)	36.6(34.1–39.3)	0.151	37.3(34.5–42.5)	37.1(34.3–42.9)	0.976
TT (s)	16.6(15.9–17.1)	16.3(15.6–17.3)	0.140	17.0(16.0–18.4)	17.4(16.2–19.4)	0.066
D-dimer (mg/L)	0.4(0.2–1.0)	1.2(0.8–3.1)	**<0.001**	0.9(0.6–2.1)	1.2(0.7–2.4)	0.056
CRP (mg/L)	2.1(0.9–5.1)	9.3(3.4–22.6)	**<0.001**	7.3(4.0–19.6)	11.0(4.0–25.4)	**0.039**
Initial treatment in hospital						
Anticoagulants, *n* (%)	48(18.3%)	74(28.2%)	**0.010**	79(49.1%)	81(50.3%)	0.911
Antiplatelets, *n* (%)	238(90.8%)	140(53.4%)	**<0.001**	105(65.2%)	91(56.5%)	0.138

HT, hemorrhagic transformation; sHT, spontaneous HT; tHT, HT after thrombectomy; TOAST, Trial of ORG 10172 in Acute Stroke Treatment; AF, atrial fibrillation; CHD, coronary heart disease; NIHSS, National Institutes of Health Stroke Scale; mRS, modified Rankin Scale; RBC, red blood cell; WBC, white blood cell; Hb, hemoglobin; PLT, platelet; ALT, alanine aminotransferase; AST, aspartate amino transferase; PT, prothrombin time; APTT, activated partial thromboplastin time; TT, thrombin time; CRP, C-reaction protein. *Continuous variables were compared between the groups by the Student’s *t*-test or the Mann–Whitney test. The chi-square test was used for categorical variables. Bold values are *p* < 0.05.

### 3.2 Baseline characteristics of fibrinogen level in the two cohorts

We divided two cohorts into three equal parts based on the distribution of fibrinogen (Supplementary Tables S2, S3): for sHT level range (0.95–1.00 μmol/L), T1 was 0.95–3.09 μmol/L, T2 was 3.10–3.95 μmol/L, and T3 was 3.96–10.00 μmol/L; for tHT level range (1.01–9.46 μmol/L), T1 was 1.01–2.71 μmol/L, T2 was 2.72–3.34 μmol/L, and T3 was 3.35–9.46 μmol/L. The demographic characteristics, vascular risk factors, laboratory test results, TOAST classification, and initial hospital treatment for the two cohorts of cases were presented using tables based on the fibrinogen trisection. The clotting time (TT) gradually decreased as fibrinogen gradually increased in both groups. A higher incidence of HT was associated with higher fibrinogen levels (*p* < 0.001) in the sHT cohort. In contrast, a higher incidence of HT was related to lower fibrinogen levels (*p* = 0.004) in the tHT cohort. The sHT cohort with high fibrinogen levels showed a higher prevalence of hyperlipidemia, platelets, white blood cell (WBC), C-reaction protein (CRP), and a lower usage rate of antiplatelet drugs compared to the subjects with low fibrinogen levels. Patients with higher fibrinogen level in the tHT cohort had a higher prevalence of hyperlipidemia, platelet count, globulin, CRP, in addition, lower PT when compared to those with lower fibrinogen levels.

### 3.3 Relationship between fibrinogen level and HT subtypes

**Figure 2:**
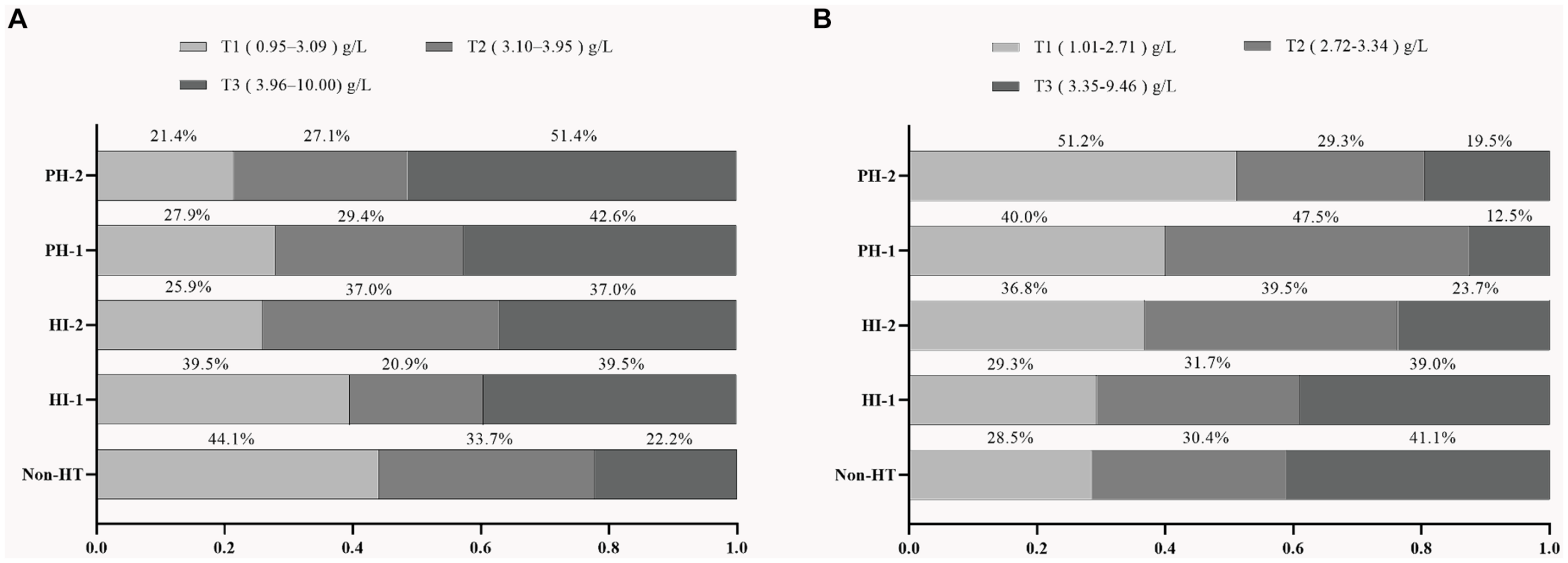
Proportion of patients in each fibrinogen trisection among AIS patients with different HT subtypes in the two cohorts. **(A)** sHT cohort, **(B)** tHT cohort. HT, hemorrhagic transformation; HI, hemorrhagic infarction; PH, parenchymal hematoma.

**Figure 3:**
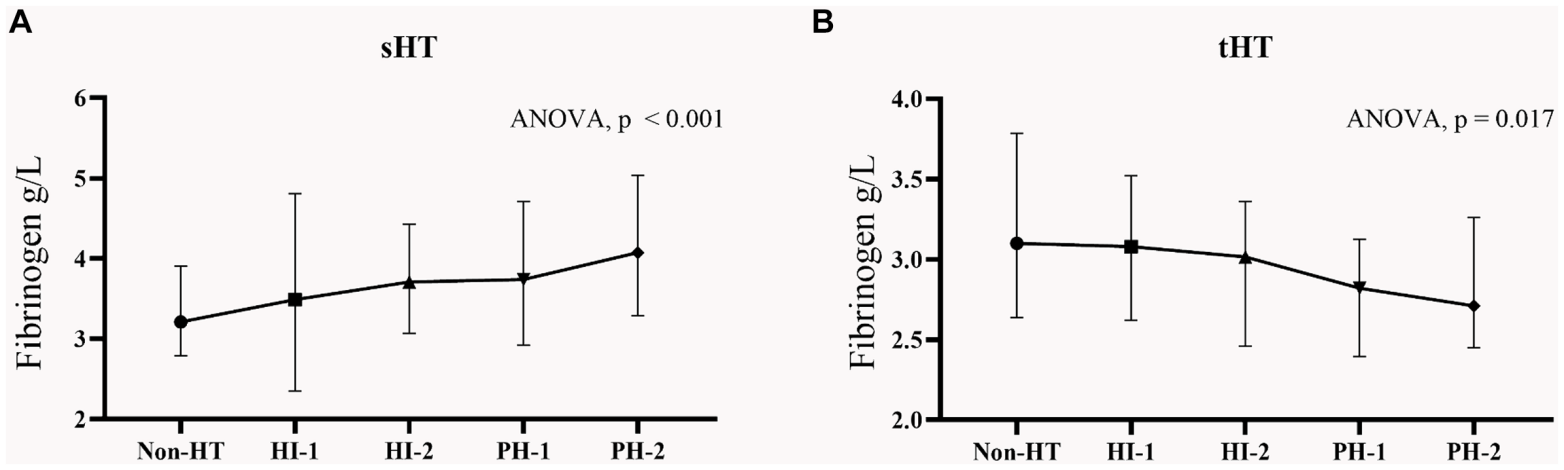
Fibrinogen concentrations in subgroups of HT in the two cohorts. Each data point and error bar correspond to the median and interquartile range of fibrinogen levels in the subgroups of HT. **(A)** sHT cohort, **(B)** tHT cohort. HT, hemorrhagic transformation; HI, hemorrhagic infarction; PH, parenchymal hematoma.

According to the percentage analysis of fibrinogen level grouping in the subtypes of HT, in the sHT cohort (Figure 2A), non-HT patients with low Fibrinogen T1 had the highest proportion (44.1%), while the PH-1 and PH-2 had the highest proportion of T3, which was higher than that of HI-1 and HI-2. In the tHT cohort (Figure 2B), non-HT patients with high Fibrinogen T3 had the highest proportion (41.1%), and PH-2 had the highest proportion of T1 (51.2%). Fibrinogen level was directly proportional to the severity of HT in sHT (Figure 3A). However, in tHT cohort, fibrinogen level was inversely proportional to the severity of HT (Figure 3B). Taking HT as the dependent variable and fibrinogen lowest trisection (T1) as the reference, the adjusted multivariate logistic regression analysis results are shown in Table 2. According to the univariate analysis (Supplementary Table S4), the unadjusted OR value of fibrinogen T3 in sHT was 3.120 (95%CI: 2.170–4.489, *p* < 0.001), and the OR value of T3 in tHT was 0.419 (95%CI: 0.263–0.666, *p* = 0.002). AF was independently correlated in sHT with an OR value of 2.408 (95%CI: 1.580–3.670, *p* = 0.001), but not statistically significant in tHT. Glucose, WBC, PLT, discharge NIHSS, admission and discharge mRS, CRP were significantly correlated with HT in both cohorts. After adjusting for confounding factors such as age, sex, history (hypertension, hyperlipidemia, diabetes, etc.), and laboratory tests (such as WBC, PLT, APTT, glucose, etc.), as well as antiplatelet and anticoagulant drugs after admission, T3 in both cohorts remained significantly and independently associated with HT risk, T3: model1: OR = 1.676 (95%CI: 1.121–2.508, *p* < 0.001), model2: OR = 2.839 (95%CI: 1.887–4.271, *p* < 0.001), model3: OR = 2.515 (95%CI: 1.339–4.725, *p* = 0.002). tHT: model1: OR = 0.417 (95%CI: 0.263–0.665, *p* = 0.002), model2: OR: 0.424 (95%CI: 0.243–0.740, *p* = 0.011), model3: OR = 0.238 (95%CI: 0.108–0.523, *p* = 0.003). Additionally, after adjusting for confounding factors, fibrinogen T2 in sHT was also significantly and independently associated with HT risk, model2: OR = 1.676 (95%CI: 1.121–2.508, *p* = 0.035), model3: OR = 2.182 (95%CI, 1.207–3.945, *p* = 0.030).

**Table 2:** Multivariate logistic regression analysis of the association between fibrinogen level and HT in the two cohorts.

	sHT cohort						tHT cohort					
	Model 1^*^		Model 2^†^		Model 3^#^		Model 1^*^		Model 2^†^		Model 3^#^	
Fibrinogen	Adjusted OR^a^ (95%CI)	*p*-value	Adjusted OR^a^ (95%CI)	*p*-value	Adjusted OR^a^ (95%CI)	*p*-value	Adjusted OR^a^ (95%CI)	*p*-value	Adjusted OR^a^ (95%CI)	*p*-value	Adjusted OR^a^ (95%CI)	*p*-value
T1	Ref		Ref		Ref		Ref		Ref		Ref	
T2	1.552 (1.073–2.245)	0.050	1.676 (1.121–2.508)	**0.035**	2.182 (1.207–3.945)	**0.030**	0.875 (0.556–1.377)	0.629	0.859 (0.494–1.493)	0.651	1.132 (0.582–2.200)	0.759
T3	3.071 (2.109–4.471)	**<0.001**	2.839 (1.887–4.271)	**<0.001**	2.515 (1.339–4.725)	**0.016**	0.417 (0.263–0.665)	**0.002**	0.424 (0.243–0.740)	**0.011**	0.238 (0.108–0.523)	**0.003**

sHT, spontaneous hemorrhage transformation; tHT, hemorrhage transformation after thrombectomy; OR, odds radio; CI, confidence level; HT, hemorrhage transformation; ^a^Reference OR (1.000) is the lowest trisection of fibrinogen for HT; ^*^Model 1: adjusted for age, sex; ^†^ Model 2: adjusted for covariates from Model 1 and further adjusted for medical history (sHT, atrial fibrillation, diabetes, hyperlipemia, hypertension, coronary heart disease, baseline NIHSS scores; tHT, atrial fibrillation, hypertension, diabetes, coronary heart disease, history of stroke, hyperlipemia and baseline NIHSS scores); ^#^Model 3 (sHT cohort): adjusted for covariates from Model 2 and further adjusted for WBC, PLT, Glucose, APTT, TT, CRP, anticoagulant, and antiplatelets; ^#^Model 3 (tHT cohort): adjusted for covariates from Model 2 and further adjusted for WBC, hemoglobin, platelet, Glucose, TT, CRP, anticoagulant and antiplatelet. Bold values are *p* < 0.05.

### 3.4 Baseline characteristics of subgroups with AF

We divided the two cohorts into two subgroups based on whether or not they had AF history (Supplementary Table S5). In the sHT cohort, HT patients without AF had higher levels of fibrinogen compared to non-HT (median 3.805 vs. 3.160, *p* < 0.001), while no difference was found between the two groups among patients with AF. The incidence of HT was higher in the cohort with a history of AF compared to the cohort without AF. In the tHT cohort, among patients without AF, those non-HT had higher levels of non-HT fibrinogen compared to those with HT (3.100 versus 2.935, *p* = 0.034), while in the AF group, non-HT fibrinogen remained higher than HT fibrinogen (3.040 versus 2.855, *p* = 0.024). Adjusted multivariate logistic regression analysis showed that in the AF-absence subgroup of sHT (Table 3), T2 and T3 were significantly and independently associated with HT risk after adjusting for confounding factors. T2: model1: OR = 1.662 (95%CI: 1.105–2.501, *p* = 0.041), model2: OR = 1.687 (95%CI: 1.089–2.611, *p* = 0.049). T3: model1: OR = 3.975 (95%CI: 2.614–6.044, *p* < 0.001), model2: OR = 3.479 (95%CI: 2.216–5.463, *p* < 0.001), model3: OR = 3.458 (95%CI: 2.010–5.947, *p* < 0.001). However, in the AF subgroup, after adjustment, any of the trisection of fibrinogen showed no significant correlation with HT risk. In tHT shown in Table 4, regardless of the presence of AF, the OR value of T3 after adjustment was significantly correlated with HT risk. In the AF-absence subgroup: T3: model1: OR = 0.446 (95%CI: 0.244–0.812, *p* = 0.027), model2: OR = 0.381 (95%CI: 0.184–0.786, *p* = 0.029), model3: OR = 0.195 (95%CI: 0.066–0.578, *p* = 0.013). In the AF-presence subgroup: model1: OR = 0.327 (95%CI: 0.122–0.877, *p* = 0.027), model3: OR = 0.161 (95%CI: 0.036–0.714, *p* = 0.044).

**Table 3:** Multivariate logistic regression analysis of the association between fibrinogen and HT with and without AF in sHT.

**sHT**	AF absence						AF presence					
	Model1^*^		Model 2^†^		Model 3^#^		Model 1^*^		Model 2^†^		Model 3^#^	
Fibrinogen	Adjusted OR^a^ (95%CI)	*p*-value	Adjusted OR^a^ (95%CI)	*p*-value	Adjusted OR^a^ (95%CI)	*p*-value	Adjusted OR^a^ (95%CI)	*p*-value	Adjusted OR^a^ (95%CI)	*p*-value	Adjusted OR^a^ (95%CI)	*p*-value
T1	Ref		Ref		Ref		Ref		Ref		Ref	
T2	1.662 (1.105–2.501)	**0.041**	1.687 (1.089–2.611)	**0.049**	1.555 (0.946–2.557)	0.144	0.783 (0.272–2.254)	0.704	1.272 (0.330–4.894)	0.769	4.397 (0.617–33.339)	0.214
T3	3.975 (2.614–6.044)	**<0.001**	3.479 (2.216–5.463)	**<0.001**	3.458 (2.010–5.947)	**<0.001**	0.559 (0.194–1.612)	0.366	0.784 (0.213–2.887)	0.758	0.825 (0.102–6.685)	0.880

sHT, spontaneous hemorrhage transformation; tHT, hemorrhage transformation after thrombectomy; OR, odds radio; CI, confidence level; HT, hemorrhage transformation; ^a^Reference OR (1.000) is the lowest trisection of fibrinogen for HT; ^*^Model 1: adjusted for age, sex; ^†^Model 2: adjusted for covariates from Model 1 and further adjusted for medical history (sHT, atrial fibrillation, diabetes, hyperlipemia, hypertension, coronary heart disease, baseline NIHSS scores); ^#^Model 3 (sHT cohort): adjusted for covariates from Model 2 and further adjusted for WBC, PLT, Glucose, APTT, TT, CRP, anticoagulant, and antiplatelet. Bold values are *p* < 0.05.

**Table 4:** Multivariate logistic regression analysis of the association between fibrinogen and HT with and without AF in tHT.

tHT	AF absence						AF presence					
	Model 1^*^		Model 2^†^		Model 3^#^		Model 1^*^		Model 2^†^		Model 3^#^	
Fibrinogen	Adjusted OR^a^ (95%CI)	*p*-value	Adjusted OR^a^ (95%CI)	*p*-value	Adjusted OR^a^ (95%CI)	*p*-value	Adjusted OR^a^ (95%CI)	*p*-value	Adjusted OR^a^ (95%CI)	*p*-value	Adjusted OR^a^ (95%CI)	*p*-value
T1	Ref		Ref		Ref		Ref		Ref		Ref	
T2	0.837 (0.462–1.515)	0.622	0.768 (0.356–1.656)	0.572	1.524 (0.560–4.151)	0.489	0.959 (0.467–1.972)	0.925	1.034 (0.427–2.501)	0.950	1.197 (0.394–3.640)	0.790
T3	0.446 (0.244–0.812)	**0.027**	0.381 (0.184–0.786)	**0.029**	0.195 (0.066–0.578)	**0.013**	0.361 (0.169–0.770)	**0.027**	0.327 (0.122–0.877)	0.062	0.161 (0.036–0.714)	**0.044**

sHT, spontaneous hemorrhage transformation; tHT, hemorrhage transformation after thrombectomy; OR, odds radio; CI, confidence level; HT, hemorrhage transformation; ^a^Reference OR (1.000) is the lowest trisection of fibrinogen for HT; ^*^Model 1: adjusted for age, sex; ^†^Model 2: adjusted for covariates from Model 1 and further adjusted for medical history (sHT, atrial fibrillation, diabetes, hyperlipemia, hypertension, coronary heart disease, baseline NIHSS scores); ^#^Model 3: adjusted for covariates from Model 2 and further adjusted for WBC, PLT, Glucose, hemoglobin, APTT, TT, CRP, anticoagulant, and antiplatelet. Bold values are *p* < 0.05.

## 4 Discussion

The study of two cohorts revealed that fibrinogen levels after admission were significantly correlated with the development of HT. The sHT cohort demonstrated a positive correlation between higher fibrinogen levels and more severe HT. Conversely, lower fibrinogen levels correlated positively with more severe HT in the tHT cohort. Specifically, the presence of AF was shown to have a significant impact on the development of HT in the sHT cohort.

Fibrinogen, a soluble 340 kDa glycoprotein, is synthesized by hepatocytes in the liver. Under normal physiological conditions, the plasma fibrinogen concentration typically ranges between 2 and 4 g/L (24). Our study is the first to compare the relationship between fibrinogen and HT in two different cohorts. We identified a positive correlation between fibrinogen levels and HT severity in sHT patients. Conversely, we observed a negative correlation between fibrinogen levels and HT severity in tHT patients, indicating distinct pathogenesis between the two cohorts.

Previous clinical studies have found that high fibrinogen is associated with the development of HT in stroke patients who have not been thrombolytic (25, 26). Delayed time to fibrinogen-to-fibrin conversion, as indicated by prolonged TT, is independently and negatively associated with spontaneous HT (27). Fibrinogen is a crucial component of the coagulation system and is linked with vasculitis disorders (28). In the presence of an intact blood–brain barrier, the central nervous system cannot detect fibrinogen. Following an acute ischemic stroke (AIS), there is a breakdown in blood–brain barrier (BBB) formation and increased permeability during reperfusion, resulting in the infiltration of fibrinogen into the central nervous system. After AIS, vascular endothelial cells are damaged, resulting in damage to the blood–brain barrier (BBB) and increased permeability during reperfusion, resulting in fibrinogen infiltration and deposition in the central nervous system (29, 30). The increased permeability during reperfusion leads to the infiltration of fibrinogen into the central nervous system. This subsequently results in the conversion of fibrinogen into fibrin, which has the potential to bind to the CD11b/CD18 integrin receptor and induce microglia activation (30). Fibrinogen can also promote the entry of inflammatory monocytes/macrophages, monocytes synthesize thromboinflammatory proteins via *de novo* synthesis (31). This mechanism has been studied in the context of multiple sclerosis (32). Moreover, fibrinogen can activate matrix metalloproteinase-9 (MMP-9) expression, which is recognized as a pro-inflammatory factor in the context of neurovascular unit injury and blood–brain barrier breakdown (33). Higher fibrinogen delays the constriction of thrombotic clots, worsens vascular occlusion, and leads to increased levels of inflammation (34). At the same time, elevated levels of inflammation biomarkers such as IL-1, IL-6, CRP, TNF-α, and blood clots contribute to the activation of inflammatory pathways (35–38). In our study, CRP levels were higher in the sHT cohort with high fibrinogen levels (T3) (11.4, 95% CI 4.2–27.5). Therefore, ischemia/reperfusion injury to tissues can contribute to blood–brain barrier dysfunction, ultimately leading to hemorrhagic transformation (39, 40).

In the sHT cohort, we found that patients who developed HT had higher levels of hyperfibrinogen, which was consistent with previous findings (41). However, it has been reported in the literature of some rt-PA treatments that low fibrinogen <1.5 g/L is associated with a high incidence of HT (42), which may be due to the significant disruption of the fibrinolytic system caused by rt-PA, and the reduction of fibrinogen in both non-HT and HT patients compared with baseline after thrombolytic therapy. Ye et al. (27) found no significant difference in fibrinogen and HT correlation, but in their study, a higher proportion of patients with a history of atrial fibrillation were higher among non-HT patients, which is consistent with our findings. Studies have shown a higher incidence of AF in the HT group than in the non-HT group, and cardioembolic strokes can be used to predict early HT (23, 43), which is consistent with our study. Our study excluded confounding factors, such as anticoagulant and antiplatelet therapy, which demonstrated that fibrinogen and HT are still independently associated. Previous reports have shown the high safety profile of anticoagulant or antiplatelet therapy for MT (44–46). Cardioembolic stroke is linked to fibrin-rich white thrombi, while non-cardioembolic cases (atherosclerotic thrombi and cryptogenic thrombi) are linked to erythrocyte-rich thrombi. Cardioembolism shows a higher ratio of fibrin to platelets than non-cardioembolism (47–49). Erythrocyte-rich thrombi can induce oxidative stress (50) through increased hemoglobin, resulting in dysregulation of heme/iron metabolism. Previous studies have found that AF is associated with more severe baseline hypoperfusion (51), leading to more severe HT (52, 53). Moreover, we found that hypertriglyceridemia was more common in patients without AF than in those with AF. Swarowska et al. (54) also concluded that persistent fibrinogen elevation in the AF group after hospitalization was associated with high triglycerides, while high triglycerides were related to systemic inflammation. But the relationship between increased fibrinogen and increased triglycerides after AIS still requires further investigation. Above findings suggest that we found no significant difference in fibrinogen levels in patients in the AF group in the non-HT and HT group. Therefore, we speculate that the HT in the subgroups of AF may be influenced by AF itself, and the predictive ability of fibrinogen in this group is influenced by AF, so that the correlation between fibrinogen and HT only suitable in sHT patients without AF. As a result, when treating stroke patients with AF, medical professionals should closely monitor their fibrinogen levels to guide clinical treatment and health management. Also, it’s worth noting that in the sHT group, 28.2% of patients received anticoagulant therapy for the first time after stroke, which was associated with 21% of patients with a history of atrial fibrillation. We found that anticoagulation therapy was a risk factor for HT (OR = 1.753, 95CI: 1.240–2.478, *p* = 0.008), while antiplatelet therapy was a protective factor for HT (OR = 0.116, 95CI: 0.077–0.175, *p* < 0.001), and we hypothesized that patients with antiplatelet therapy had a lower proportion of cardioembolism (26), while thrombin was a key factor in the conversion of soluble fibrinogen to insoluble fibrin, which would be elevated when anticoagulation was taken.

In patients with tHT, studies have found a decrease in fibrinogen levels after thrombectomy, and lower fibrinogen levels predict a higher risk of HT (13, 55). The mechanism of fibrinogen production may be attributed to surgical procedures that lead to the accumulation of fibrinogen in blood vessels, forming a thrombus. Additionally, physical instruments used to stretch and compress blood vessels may cause vascular endothelium damage (56) or vessel occlusion (57), initiating a series of reactions, such as platelet activation, coagulation factor release, and vasoconstriction (28, 58). These reactions increase the risk of fibrinogen buildup on the blood vessel walls, forming a stable thrombus that is subsequently depleted (59). Hemodilution during surgery may cause a decrease in fibrinogen (60). Besides, anticoagulant treatment may be applied during thrombectomy to prevent thrombosis, which can also impact fibrinogen synthesis and release. Studies on fibrinogen in patients undergoing thrombolysis have shown that fibrinogen depletion after AIS might lead to potential hemorrhagic infarcts converting into PH or an increase in the volume of PH, ultimately increasing the incidence of HT (61, 62). During cardiac surgery, postoperative fibrinogen-fibrin products are elevated (63) due to intraoperative extracorporeal circulation hemodilution and clotting factor depletion (64), postoperative fibrinogen levels are an independent risk factor for excessive hemorrhage, and decreased fibrinogen levels increase the risk of postoperative hemorrhage (59, 65, 66). Suitable levels of fibrinogen are necessary to facilitate effective thrombosis and prevent HT consequences.

The highlight of the study is that for the first time, we found that fibrinogen levels in patients with atrial fibrillation were positively correlated with the development of HT in the sHT group, but showed an inverse relationship in the tHT cohort, which has not been demonstrated in other studies. These findings suggest that fibrinogen levels on admission may help clinicians identify patients at increased risk of HT, and that for patients not receiving reperfusion therapy, timely detection and intervention of high fibrinogen levels can reduce the risk of HT development, while for patients undergoing MT surgery, low fibrinogen levels should be avoided.

Our study has some limitations. Firstly, it was a retrospective, single-center study, we could not establish the exact causality, and fibrinogen levels were only measured only once after admission, and future studies need to investigate the fibrinogen dynamics during hospitalization, fibrinogen may be measured at different concentrations at different times of detection (67), and Lip et al. (68) found that fibrinogen peaked 1 week after stroke, but Tamam et al. (69) found that h-CRP and fibrinogen reached their highest values on day 3 after stroke. Secondly, baseline imaging data, such as infarct area and site, were not included, even though they potentially affect the occurrence and development of hemorrhagic transformation. Thirdly, patients who underwent thrombolysis-alone and bridging treatments were excluded, and the comparison of fibrinogen levels with HT in these two cohorts needs further exploration. In addition, we did not include inflammatory factors such as IL-1 and IL-6, and analyzed the relationship between fibrinogen levels and them. Although we adjusted for confounders, we had to admit that gender, age, diabetes, hypertension, lipids, and initial stroke severity were all associated with HT (5, 33). In MT surgery, the occurrence and severity of HT are also related to factors such as surgical time (70), the method of clot retrieval (71), thrombectomy passes and others. Thrombosis in fibrin-rich tissues has been found to be associated with a higher number of recanalization operations and a longer recanalization time required during surgery (48, 72).

## 5 Conclusion

In conclusion, for sHT patients with non-AF, high fibrinogen levels are associated with the incidence of HT, while in tHT patients, there is a correlation between low fibrinogen and the occurrence of HT.

## Data availability statement

The raw data supporting the conclusions of this article will be made available by the authors, without undue reservation.

## Ethics statement

The study involving humans was approved by The Institutional Review Board and Ethics Committee of the First Affiliated Hospital of Wenzhou Medical University. Informed consent was waived since all data were anonymized and this was a retrospective study.

## Author contributions

JL: Data curation, Writing – original draft. JC: Data curation, Software, Writing – review & editing. GH: Conceptualization, Writing – review & editing. ZC: Data curation, Writing – review & editing. HZ: Data curation, Writing – review & editing. YZ: Data curation, Writing – review & editing. QD: Data curation, Writing – review & editing. BW: Data curation, Writing – review & editing. JH: Investigation, Project administration, Writing – review & editing.
