# Supplementary material for: The differences of fibrinogen levels in various types of hemorrhagic transformations
Source: Front Neurol. 2024 Jul 25;15:1364875. doi: 10.3389/fneur.2024.1364875 (PMC11306044; doi:10.3389/fneur.2024.1364875)
Supplement: Supplementary file 1 [file Data_Sheet_1.docx]

**Supplement material**

**Title: The differences of fibrinogen levels in various types of hemorrhagic transformations**

Jingfang Long^1,2†^, Jiahao Chen ^1†^, Guiqian Huang^1,3†^, Zhen Chen^4†^, Heyu Zhang^1^, Ye Zhang^1^, Qi Duan^1^, Beilan Wu^1^, Jincai He^1^*

^1^ Department of Neurology, the First Affiliated Hospital of Wenzhou Medical University, Wenzhou 325000, Zhejiang, China

^2^ Department of Neurology, Wenzhou Central Hospital, Wenzhou, 325000, Zhejiang, China

^3^ School of Mental Health, Wenzhou Medical University, Wenzhou, 325000, Zhejiang, China

^4^ Zhejiang Provincial Key Laboratory of Aging and Neurological Disorder Research, The First Affiliated Hospital of Wenzhou Medical University, Wenzhou 325000, Zhejiang, China

**^†^** These authors contributed equally to this work.

**^*^Corresponding author**: Jincai He

JinCai He, M.D.

Department of Neurology, the First Affiliated Hospital of Wenzhou Medical University, Wenzhou, 325000, Zhejiang, China；

E-mail : [hjc@wmu.edu.cn](mailto:hjc@wmu.edu.cn) fax : (86) 577-55578999 ; tel : (86) 577-55578999

Table S1 Differences of Baseline Characteristics in AIS patients in Two Cohorts

| Variables | sHT(n=524) | tHT(n=322) | p-value |
| --- | --- | --- | --- |
| **Demographic Parameters** | |  |  |
| Age (years) | 67(59-74) | 70(61-77) | **<0.001** |
| Sex (Male, n%) | 373(71.2%) | 222(68.9%) | 0.539 |
| **Vascular risk factors** | | | |
| TOAST |  |  | **<0.001** |
| 1 | 443(84.5%) | 149(46.3%) |  |
| 2 | 61(11.6%) | 138(42.9%) |  |
| 3 | 20(3.8%) | 35(10.9%) |  |
| History of AF, n (%) | 81(15.5%) | 127(39.4%) | **<0.001** |
| History of hypertension, n (%) | 322(61.5%) | 220(68.3%) | 0.051 |
| History of diabetes, n (%) | 142(27.1%) | 69(21.4%) | 0.077 |
| History of hyperlipemia, n (%) | 39(7.4%) | 187(58.1%) | **<0.001** |
| History of CHD, n (%) | 46(8.8%) | 40(12.4%) | 0.113 |
| History of stroke, n (%) | 49(9.4%) | 43(13.4%) | 0.089 |
| Current smoking, n (%) | 177(33.8%) | 123(38.2%) | 0.218 |
| Current drinking, n (%) | 152(29.0%) | 107(33.2%) | 0.224 |
| mRS on admission, median (IQR) | 2(2-3) | 5(4-5) | **<0.001** |
| mRS on discharge, median (IQR) | 2(1-3) | 4(3-5) | **<0.001** |
| NIHSS on admission, median (IQR) | 7(3-11) | 16(11-22) | **<0.001** |
| NIHSS on discharge, median (IQR) | 5(1-10) | 10(4-19) | **<0.001** |
| **Biochemistry and vital signs on admission** | | | |
| RBC (×10^12^/L) | 4.5(4.2-4.8） | 4.1(3.6-4.5) | **<0.001** |
| WBC (×10^9^/L) | 7.4(6.0-8.8) | 9.6(7.6-11.8) | **<0.001** |
| Hb (g/L) | 137.0(127.0-146.0) | 124.5(111.0-138.0) | **<0.001** |
| PLT(×10^9^/L) | 194.0(162.0-230.0) | 182.5(149.0-234.8) | 0.120 |
| Glucose (mmol/L) | 5.4(4.6-6.9) | 7.0(5.8-8.9) | **<0.001** |
| Albumin (g/L) | 37.6(35.6-40.1) | 36.4(31.4-37.5) | **<0.001** |
| Globulin (g/L) | 29.4(26.8-32.7) | 26.3(24.1-29.2) | **<0.001** |
| ALT (U/L) | 19.0(14.0-28.0) | 17.0(12.0-26.0) | 0.024 |
| AST (U/L) | 23.0(19.0-32.0) | 24.0(19.3-30.0) | 0.907 |
| Fibrinogen | 3.45(2.90-4.32) | 2.98(2.57-3.57) | **<0.001** |
| PT (s) | 137.0(127.0-146.0) | 124.5(111.0-138.0) | **<0.001** |
| APTT (s) | 36.3(34.1-38.8) | 37.2(34.3-42.6) | **<0.001** |
| TT (s) | 16.5(15.7-17.15 | 17.2(16.0-18.8) | **<0.001** |
| D-dimer (mg/L) | 1.1(0.6-2.7) | 1.1(0.6-2.3) | 0.756 |
| CRP (mg/L) | 3.2(1.1-10.1) | 8.2(4.0-23.3) | **<0.001** |
| **Initial treatment in hospital** | | | |
| Anticoagulants, n (%) | 122(23.3%) | 160(49.7%) | **<0.001** |
| Antiplatelets, n (%) | 378(72.1%) | 196(60.1%) | **<0.001** |

NOTE: HT: hemorrhagic transformation; sHT: spontaneous HT; tHT: HT after thrombectomy; TOAST: Trial of ORG 10172 in Acute Stroke Treatment; AF: atrial fibrillation; CHD: coronary heart disease; NIHSS: National Institutes of Health Stroke Scale; mRS: modified Rankin Scale; RBC: red blood cell; WBC: white blood cell; Hb: hemoglobin; PLT: platelet; PT: prothrombin time; APTT: activated partial thromboplastin time; TT: thrombin time; CRP: C-reaction protein.* Continuous variables were compared between the groups by the Student’s t-test or the Mann–Whitney test. The chi-square test was used for categorical variables.

Table S2 Baseline Characteristics of the AIS Patients in the sHT Cohort According to fibrinogen Trisection

|  | | Fibrinogen Trisection | | | |
| --- | --- | --- | --- | --- | --- |
| Variables | All Patients | Trisection 1 n = 186 (0.95–3.09 g/L) | Trisection 2 n = 166 (3.10–3.95 g/L) | Trisection 3 n = 170 (3.96–10.00 g/L) | P value * |
| HT, n (%) |  | 71(38.2%) | 78(47.0%) | 112(65.9%) | **<0.001** |
| **Demographic Parameters** | | | |  |  |
| Age (years) | 67(59-74) | 65(58-73) | 68(59-74) | 70(60-75) | 0.078 |
| Sex (Male, n%) | 373(71.2%) | 134(72.0%) | 115(69.3%) | 123(72.4%) | 0.789 |
| **Vascular risk factors** | | | |  |  |
| TOAST, n (%) |  |  |  |  | 0.069 |
| Large artery | 443(84.5%) | 159(85.5%) | 135(81.3%) | 147(86.5%) |  |
| Cardioembolism | 61(11.6%) | 21(11.3%) | 19(11.4%) | 21(15.3%) |  |
| Others | 20(3.8%) | 6(3.2%) | 21(12.7%) | 2 |  |
| History of AF, n (%) | 81(15.5%) | 23(12.4%) | 28(16.8%) | 30(18%) | 0.328 |
| History of hypertension, n (%) | 322(61.5%) | 105(56.5%) | 104(62.7%) | 111(65.3%) | 0.211 |
| History of diabetes, n (%) | 142(27.1%) | 55(11.3%) | 42(25.3%) | 44(1.2%) | 0.615 |
| History of hyperlipemia，n (%) | 39(7.4%) | 11(5.9%) | 13(7.8%) | 15(8.8%) | 0.567 |
| History of CHD, n (%) | 46(8.8%) | 16(8.6%) | 13(7.8%) | 16(9.4%) | 0.875 |
| History of stroke, n (%) | 49(9.4%) | 13(11.3%) | 20(12.0%) | 15(8.8%) | 0.255 |
| Current smoking, n (%) | 177(33.8%) | 62(33.3%) | 60(36.1%) | 55(32.4%) | 0.748 |
| Current drinking, n (%) | 152(29.0%) | 62(33.3%) | 44(26.5%) | 46(27.1%) | 0.287 |
| mRS on admission, median (IQR) | 2(2-3) | 2(2-3) | 2(1-3) | 3(2-4) | 0.060 |
| mRS on discharge, median (IQR) | 2(1-3) | 2(1-3) | 2(1-3) | 2(1-3) | 0.819 |
| NIHSS on admission, median (IQR) | 7(3-11) | 6(3-10) | 5(2-10) | 8(4-11) | **0.006** |
| NIHSS on discharge, median (IQR) | 5(1-10) | 4(1-11) | 4(1-10) | 6(2-11) | 0.112 |
| **Biochemistry and vital signs on admission** | | |  |  |  |
| RBC (×10^12^/L) | 4.5(4.2-4.8） | 4.4(4.2-4.8) | 4.5(4.2-4.9) | 4.5(4.1-4.8) | 0.617 |
| WBC (×10^9^/L) | 7.4(6.0-8.8) | 7.3(5.7-8.5) | 7.2(5.9-8.3) | 7.7(6.5-9.4) | **0.002** |
| Hb (g/L) | 137.0(127.0-146.0) | 138(128.0-146.0) | 138.0(128.0-148.0) | 136.0(126.0-145.0) | 0.384 |
| PLT(×10^9^/L) | 194.0(162.0-230.0) | 191.0(156.0-229.0) | 190.0(163.0-228.0) | 198.0(164.0-237.0) | 0.251 |
| Glucose (mmol/L) | 5.4(4.6-6.9) | 5.0(4.5-7.1) | 5.3(4.6-6.5) | 5.7(4.8-6.8) | 0.143 |
| Albumin (g/L) | 37.6(35.6-40.1) | 38.3(36.3-40.4) | 38.1(36.2-40.7) | 36.7(24.5-39.5) | **<0.001** |
| Globulin (g/L) | 29.4(26.8-32.7) | 28.3(25.6-30.8) | 29.1(27.0-31.9) | 31.7(27.9-34.7) | **<0.001** |
| ALT (U/L) | 19.0(14.0-28.0) | 19.0(14.0-28.0) | 19.0(13-27) | 21.0(13.0-29.0) | 0.748 |
| AST (U/L) | 23.0(19.0-32.0) | 23.0(19.0-30.0) | 22.0(19-30) | 26.0(21.0-34.0) | **0.011** |
| PT (s) | 137.0(127.0-146.0) | 13.5(13.0-14.2) | 13.4(12.9-14.0) | 13.5(13.1-14.2) | 0.243 |
| APTT (s) | 36.3(34.1-38.8) | 35.8(33.8-38.3) | 36.3(33.9-38.9) | 36.9(35.0-39.4) | **0.017** |
| TT (s) | 16.5(15.7-17.15 | 16.9(16.4-17.8) | 16.3(15.7-16.9) | 16.0(15.4-16.8) | **<0.001** |
| D-dimer (mg/L) | 1.1(0.6-2.7) | 1.0(0.4-3.5) | 0.9(0.5-2.0) | 1.2(0.7-3.0) | 0.317 |
| CRP (mg/L) | 3.2(1.1-10.1) | 1.5(0.7-3.6) | 3.1(1.2-6.1) | 11.4(4.2-27.5) | **<0.001** |
| **Initial treatment in hospital** | | |  |  |  |
| Anticoagulants, n (%) | 122(23.3%) | 33(17.7%) | 45(27.1%) | 44(25.9%) | 0.075 |
| Antiplatelets, n (%) | 378(72.1%) | 146(78.5%) | 123(74.1%) | 107(62.9%) | **0.004** |

NOTE: HT: hemorrhagic transformation; sHT: spontaneous HT; tHT: HT after thrombectomy; TOAST: Trial of ORG 10172 in Acute Stroke Treatment; AF: atrial fibrillation; CHD: coronary heart disease; NIHSS: National Institutes of Health Stroke Scale; mRS: modified Rankin Scale; RBC: red blood cell; WBC: white blood cell; Hb: hemoglobin; PLT: platelet; ALT: alanine aminotransferase; AST: spartate amino transferase; PT: prothrombin time; APTT: activated partial thromboplastin time; TT: thrombin time; CRP: C-reaction protein.* Continuous variables were compared between the groups by the Student’s t-test or the Mann–Whitney test. The chi-square test was used for categorical variables.

Table S3 Baseline Characteristics of the AIS Patients in the tHT Cohort According to fibrinogen Trisection

|  | | Fibrinogen Trisection | | | |
| --- | --- | --- | --- | --- | --- |
| Variables | All Patients | Trisection 1 n = 108 (1.01–2.71 g/L ) | Trisection 2 n = 107 (2.72–3.34 g/L ) | Trisection 3 n =103 (3.35–9.46 g/L ) | p-Value * |
| HT, n (%) | 160(50.3%) | 63(58.3%） | 59(55.1%) | 38(36.9%) | **0.004** |
| **Demographic Parameters** | | | | | |
| Age (years) | 70(61-77) | 72(61-76) | 69(63-75) | 70(59-78) | 0.761 |
| Sex (Male, n%) | 222(68.9%) | 31(28.7%) | 38(35.6%) | 28(27.2) | 0.374 |
| **Vascular risk factors** | | | | | |
| TOAST ,n(%) |  |  |  |  | 0.298 |
| Large artery | 149(46.3%) | 41(38.0%) | 54(50.5%) | 52(50.5%) |  |
| Cardioembolism | 138(42.9%) | 54(50.0%) | 43(40.2%) | 39(37.9%) |  |
| Others | 35(10.9%) | 13(12.0%) | 10(9.3%) | 12(11.7%) |  |
| History of AF, n(%) | 127(39.4%) | 48(44.4%) | 41(38.1%) | 36(35.0%) | 0.357 |
| History of hypertension, n (%) | 220(68.3%) | 66(61.1%) | 76(71.0%) | 76(73.8%) | 0.112 |
| History of diabetes, n (%) | 69(21.4%) | 18(16.7%) | 28(26.2) | 22(21.4%) | 0.236 |
| History of hyperlipemia, n (%) | 187(58.1%) | 54(50.0%) | 60(56.1%) | 71(68.9%) | **0.025** |
| History of CHD, n (%) | 40(12.4%) | 14(13.0%) | 17(15.9%) | 8(7.8%) | 0.193 |
| History of stroke, n (%) | 43(13.4%) | 11(10.2%) | 20(18.7%) | 12(11.7%) | 0.151 |
| Current smoking, n (%) | 123(38.2%) | 40(37.0%) | 35(32.7%) | 47(45.6%) | 0.148 |
| Current drinking, n (%) | 107(33.2%) | 40(37.0%) | 31(29.0%) | 35(34.0%) | 0.449 |
| mRS on admission, median (IQR) | 5(4-5) | 5(4-5) | 5(4-5) | 4(4-5) | **0.033** |
| mRS on discharge, median (IQR) | 4(3-5) | 4(3-5) | 4(3-5) | 4(2.5-5) | 0.124 |
| NIHSS on admission, median (IQR) | 16(11-22) | 16(12-22) | 16(12-2) | 15(9.5-20) | 0.062 |
| NIHSS on discharge, median (IQR) | 10(4-19) | 10(4-18) | 13(7-24) | 8(3-16.5) | 0.058 |
| **Biochemistry and vital signs on admission** | | | | | |
| RBC (×10^12^/L) | 4.1±0.62 | 4.02±0.64 | 4.13±0.54 | 4.03±0.66 | 0.385 |
| WBC (×10^9^/L) | 9.6(7.6-11.8) | 9.2(7.5-11.3) | 10.0(7.9-11.9) | 9.8(7.6-12.3) | 0.536 |
| Hb (g/L) | 124.5(111.0-138.0) | 124(112-138) | 128.0(113.5-138.0) | 123.0(108.0-137.5) | 0.315 |
| PLT(×10^9^/L) | 182.5(149.0-234.8) | 168.5(141.5-201) | 182.0(151.5-220.5) | 217.0(161.5-273.5) | **<0.001** |
| Glucose (mmol/L) | 7.0(5.8-8.9) | 7.3(5.9-9.2) | 7.1(5.7-9.2) | 6.7(5.8-8.4) | 0.676 |
| Albumin (g/L) | 36.4(31.4-37.5) | 34.8(31.7-37.8) | 34.2(31.5-37.4) | 34.8(31.2-37.5) | 0.876 |
| Globulin (g/L) | 26.3(24.1-29.2) | 25.6(23.4-28.3) | 26.1(24.1-28.4) | 27.9(25.0-30.7) | **<0.001** |
| ALT (U/L) | 17.0(12.0-26.0) | 16.5(12.0-24.5) | 18.5(13.0-31.5) | 17.0(12.0-24.5) | 0.501 |
| AST (U/L) | 24.0(19.3-30.0) | 24.0(20.0-30.0) | 24.0(20.0-34.0) | 32.0(19.0-29.0) | 0.545 |
| PT (s) | 124.5(111.0-138.0) | 14.3(13.7-15.2) | 13.9(13.2-14.5) | 14.0(13.2-14.6) | **0.008** |
| APTT (s) | 37.2(34.3-42.6) | 37.3(33.6-41.4) | 36.4(34.3-44.8) | 38.3(35.3-43.8) | 0.313 |
| TT (s) | 17.2(16.0-18.8) | 18.0(16.7-19.8) | 16.9(15.9-18.8) | 16.6(15.5-17.9) | **<0.001** |
| D-dimer (mg/L) | 1.1(0.6-2.3) | 1.13(0.51-2.75) | 1.02(0.7-2.1) | 1.3(0.7-2.1) | 0.705 |
| CRP (mg/L) | 8.2(4.0-23.3) | 5.2(3.2-15.35) | 6.9(4.0-18.6) | 15.3(6.5-34.8) | **<0.001** |
| **Initial treatment in hospital** | | |  |  |  |
| Anticoagulants, n (%) | 160(49.7%) | 57(52.8%) | 52(48.6%) | 48(46.6%) | 0.656 |
| Antiplatelets, n (%) | 196(60.1%) | 56(51.9%) | 70(65.4%) | 68(66.0%) | 0.059 |

NOTE: HT: hemorrhagic transformation; sHT: spontaneous HT; tHT: HT after thrombectomy; TOAST: Trial of ORG 10172 in Acute Stroke Treatment; AF: atrial fibrillation; CHD: coronary heart disease; NIHSS: National Institutes of Health Stroke Scale; mRS: modified Rankin Scale; RBC: red blood cell; WBC: white blood cell; Hb: hemoglobin; PLT: platelet; ALT: alanine aminotransferase; AST: spartate amino transferase; PT: prothrombin time; APTT: activated partial thromboplastin time; TT: thrombin time; CRP: C-reaction protein.* Continuous variables were compared between the groups by the Student’s t-test or the Mann–Whitney test. The chi-square test was used for categorical variables

Table S4 Unvariate Analysis of AIS patients in Two Cohorts

|  | sHT | | tHT | |
| --- | --- | --- | --- | --- |
| Variables | OR(95%CI) | P-value | OR(95%CI) | P-value |
| Fibrinogen | 1.548(1.350- 1.774） | **<0.001** | 0.676(0.552-0.828) | **0.001** |
| T1 |  |  |  |  |
| T2 | 1.435(1.005-2.048) | 0.096 | 0.878(0.559-1.381) | 0.637 |
| T3 | 3.120(2.170-4.489) | **<0.001** | 0.4188(0.263-0.666) | **0.002** |
| Age | 0.990(0.976-1.005) | 0.274 | 1.001(0.986-1.017) | 0.910 |
| Sex | 0.981(0.715-1.348) | 0.923 | 1.060(0.713-1.574) | 0.810 |
| History of hypertension | 0.968(0.721-1.300) | 0.858 | 0.945(0.637-1.400) | 0.811 |
| History of hyperlipemia | 1.057(0.611-1.827) | 0.868 | 0.843(0.576-1.233) | 0.459 |
| History of diabetes | 0.962(0.687-1.330) | 0.844 | 1.202(0.769-1.880) | 0.498 |
| History of AF | 2.408(1.580-3.670) | **<0.001** | 1.199(0.824-1.745) | 0.426 |
| History of CHD | 1.000(0.602-1.661) | 1 | 1.121(0.643-1.953) | 0.736 |
| NIHSS on admission | 1.168(1.129-1.209) | **<0.001** | 1.027(1.000-1.056) | 0.104 |
| NIHSS on discharge | 1.094(1.062-1.127) | **<0.001** | 1.057(1.034-1.080) | **<0.001** |
| mRS on admission | 1.860(1.618-2.138) | **<0.001** | 1.392(1.131-1.713) | **0.009** |
| mRS on discharge, median | 1.389(1.237-1.561) | **<0.001** | 1.388(1.214-1.586) | **<0.001** |
| RBC | 0.830(0.638-1.079) | 0.242 | 1.353(1.001-1.827) | 0.098 |
| WBC | 1.139(1.063-1.219) | **0.002** | 1.110(1.047-1.176) | **0.003** |
| Hb | 0.990(0.981-0.999) | 0.064 | 1.010(1.001-1.019) | 0.074 |
| PLT | 0.996(0.994-0.999) | **0.023** | 0.996(0.994-0.999) | **0.024** |
| Glucose | 1.167(1.091-1.245) | **<0.001** | 1.161(1.085-1.242) | **<0.001** |
| Albumin | 0.923(0.889-0.960) | **<0.001** | 0.935(0.898-0.974) | **0.007** |
| Globulin | 1.026(0.100-1.052) | 0.101 | 0.955(0.911-1.001) | 0.106 |
| PT | 1.639(1.386-1.937) | **<0.001** | 1.076(0.959-1.209) | 0.296 |
| APTT | 0.9997(0.9986-1.0008) | 0.663 | 1.012(1.001-1.023) | 0.072 |
| TT | 1.013(0.982-1.045) | 0.480 | 1.015(1.005-1.025) | **0.011** |
| CRP | 1.035(1.022-1.049) | **<0.001** | 1.015(1.005-1.024) | **0.012** |
| D-Dimer | 0.990(0.971-1.009) | 0.372 | 1.041(0.982-1.103) | 0.256 |
| Anticoagulant | 1.753(1.240-2.478) | **0.008** | 1.051(0.729-1.515) | 0.824 |
| Antiplatelet | 0.116(0.077-0.175) | **<0.001** | 0.694(0.476-1.012) | 0.111 |

NOTE: HT: hemorrhagic transformation; sHT: spontaneous HT; tHT: HT after thrombectomy; TOAST: Trial of ORG 10172 in Acute Stroke Treatment; AF: atrial fibrillation; CHD: coronary heart disease; NIHSS: National Institutes of Health Stroke Scale; mRS: modified Rankin Scale; RBC: red blood cell; WBC: white blood cell; Hb: hemoglobin; PLT: platelet; PT: prothrombin time; APTT: activated partial thromboplastin time; TT: thrombin time; CRP: C-reaction protein

Table S5 Differences of the baseline characteristics in AIS patients with and without AF in two cohorts

|  | sHT | | | | | | tHT | | | | | |
| --- | --- | --- | --- | --- | --- | --- | --- | --- | --- | --- | --- | --- |
|  | AF absence | | | AF presence | | | AF absence | | | AF presence | | |
| Variables | Non-HT (n=236) | HT  (n=207) | P-value | Non-HT  (n=26) | HT  (n=55) | P-value | Non-HT  (n=101) | HT  (n=94) | P-value | Non-HT  (n=60) | HT  (n=67) | P-value |
| **Demographic Parameters** | | | | | | |  |  |  |  |  |  |
| Age (years) | 67(56-73) | 66(58-75) | 0.976 | 72(68-76) | 68(60-75) | 0.341 | 67.0(56-75) | 67.5(59-77) | 0.601 | 74(69-78) | 72(66-78) | 0.262 |
| Sex (Male, n%) | 170(70.0%) | 150(72.5%) | 1 | 16(61.5%) | 37(67.2%) | 0.798 | 78(77.2%) | 23(24.5%) | 0.342 | 34(56.7%) | 44(65.7%) | 0.391 |
| TOAST, n (%) |  |  | 0.354 |  |  | **0.008** |  |  | 0.605 |  |  | 0.215 |
| Large artery | 222(94.1%) | 201(97.1%) |  | 1(3.8%) | 19(34.5%) |  | 72(71.3%) | 62(66.0%) |  | 8(13.3%) | 7(10.4%) |  |
| Cardioembolism | 2(0.8%) | 0(0%) |  | 24(92.3%) | 35(63.6) |  | 15(14.9%) | 19(20.2%) |  | 46(76.7%) | 58(86.6%) ( |  |
| Others | 12(5.1%) | 6(2.9%) |  | 1(3.8%) | 1(1.8%) |  | 14(13.9%) | 13(13.8%) |  | 6(10.0%) | 2(3.0%) |  |
| History of hypertension, n (%) | 146(61.9%) | 121(58.5%) | 0.526 | 16(61.5%) | 39(70.9%) | 0.556 | 70(69.3%) | 61(64.9%) | 0.615 | 41(68.3%) | 48(71.6%) | 0.832 |
| History of diabetes, n (%) | 66(28.0%) | 49(23.7%) | 0.358 | 6(23.1%) | 21(38.1%) | 0.274 | 17(16.8%) | 19(20.2%) | 0.672 | 15(25.0%) | 18(26.9%) | 0.971 |
| History of hyperlipemia | 17(7.2%) | 15(7.2%) | 1.000 | 2(7.7%) | 5(9.1%) | 1.000 | 64(63.4%) | 58(61.7%) | 0.862 | 33(55.0%) | 32(47.8%) | 0.624 |
| History of CHD, n (%) | 19(8.1) | 20(9.7%) | 0.668 | 4(15.4%) | 3(5.5%) | 0.289 | 10(9.9%) | 6(6.4%) | 0.527 | 9(15.0%) | 15(22.4%) | 0.404 |
| Current smoking, n (%) | 69(29.2%) | 87(42.0%) | 0.499 | 2(7.7%) | 19(34.5%) | **0.021** | 48(47.5%) | 38(40.4%) | 0.393 | 20(33.3%) | 17(25.4%) | 1.000 |
| Current drinking, n (%) | 66(28.0%) | 63(30.4%) | 0.641 | 7(26.9%) | 16(29.1%) | 1.000 | 34(33.7%) | 37(39.4%) | 0.498 | 19(31.6%) | 17(25.4%) | 0.556 |
| mRS on admission, median (IQR) | 2(1-3) | 3(2-4) | **<0.001** | 2(1-3) | 3(2-4) | **0.008** | 4(3-5) | 5(4-5) | **0.002** | 5(4-5) | 5(4-5) | 0.375 |
| mRS on discharge, median (IQR) | 2(1-3) | 2(1-3) | **<0.001** | 1(1-2) | 2(1-3) | **0.013** | 4(1-5) | 4(3.25-5) | **<0.001** | 4(2.75-5) | 5(4-5) | 0.110 |
| NIHSS on admission, median (IQR) | 4(2-8) | 9(5-13) | **<0.001** | 3(1-10) | 7(4-11) | **0.041** | 14.6±7.07 | 17.1±8.08 | **0.042** | 17.6±7.12 | 17.4±7.51 | 0.907 |
| NIHSS on discharge, median (IQR) | 3(1-10) | 8(3-11) | **<0.001** | 2(1-8) | 5(3-11) | 0.074 | 7.5(2-15) | 15(7-26) | **<0.001** | 9(3.5-15) | 12(7.5-21) | **0.040** |
| **Biochemistry and vital signs on admission** | | | | | | | | | | | | |
| RBC (×10^12^/L) | 4.5(4.2-4.8) | 4.4(4.12-4.78) | 0.420 | 4.5(4.0-5.0) | 4.4(4.0-4.8) | 0.436 | 4.0±0.61 | 4.2±0.55 | 0.081 | 4.0±0.65 | 4.0±0.68 | 0.496 |
| WBC (×10^9^/L) | 7.3(5.9-6.1) | 7.41(6.12-9.47) | 0.075 | 6.8(5.9-8.0) | 7.6(6.2-9.5) | 0.169 | 9.3(7.4-11.1) | 10.4(8.9-13.1) | **0.002** | 8.4(6.8-11) | 9.8(7.6-11.9) | 0.055 |
| Hb (g/L) | 138.0(128-146) | 136(127-145) | 0.320 | 140.0(130.0-150.0) | 135.0(126.0-145.0) | 0.172 | 124.0(110.0-135.0) | 130.0(114.5-142.0) | **0.027** | 124(107.5-132.3) | 122.0(112.0-137.5) | 0.403 |
| PLT(×10^9^/L) | 201.0(174.0-234.0) | 187(152-232) | **0.006** | 183±40.76 | 182±51.60 | 0.940 | 191.0(162.0-238.0) | 186.5(158.3-230.8) | 0.734 | 188.0(147.0-246.0) | 161.0(138.0-201.0) | **0.016** |
| Glucose (mmol/L) | 5.0(4.4-6.5) | 5.6(4.7-6.9) | **0.001** | 5.3(4.7-6.1) | 6.1(5.3-8.1) | **0.006** | 6.3(5.4-7.7) | 7.8(6.4-10.4) | **<0.001** | 6.7(5.7-9.4) | 7.7(6.1-9.6) | 0.123 |
| Fibrinogen(g/L) | 3.16(2.79-3.81) | 3.81(3.06-4.74) | **<0.001** | 3.76(2.99-4.41) | 3.51(2.98-4.52) | 0.919 | 3.10(2.69-7.75) | 2.94(2.45-3.41) | **0.034** | 3.04(2.63-3.92) | 2.86(2.46-3.16) | **0.024** |
| PT (s) | 13.3(12.8-13.7) | 13.7(13.1-14.4) | **0.001** | 13.9(13.3-14.2) | 13.9(13.5-14.7) | 0.183 | 13.8(132.0-14.4) | 14.0(13.3-14.6) | 0.284 | 14.1(13.8-15.2) | 14.5(13.6-15.3) | 0.608 |
| APTT (s) | 36.0(34.3-38.3) | 36.6(34.1-38.8) | 0.238 | 36.7(34.3-38.4) | 37.5(34.0-40.8) | 0.634 | 36.7(33.9-42.2) | 36.0(33.9-40.0) | 0.395 | 39.2(35.2-42.5) | 39.2(35.8-47.0) | 0.296 |
| TT (s) | 16.5(15.9-17.1) | 16.3(15.5-17.1) | 0.051 | 16.6(16.1-17.4) | 16.6(16.0-17.6) | 0.919 | 16.6(15.5-18.1) | 17.1(15.9-18.8) | 0.066 | 17.7(16.6-18.8) | 17.8(16.4-22.3) | 0.583 |
| D-dimer (mg/L) | 0.3(0.2-0.7) | 1.2(0.8-3.3) | **<0.001** | 1.2 (1.1-2.1) | 1.2 (0.8-2.9) | 0.746 | 0.9(0.5-2.2) | 1.2(0.6-2.2) | 0.323 | 0.8(0.5-2.0) | 1.2(0.8-2.8) | 0.056 |
| CRP (mg/L) | 2.0(0.8-4.7) | 6.7(2.7-16.8) | **<0.001** | 2.5(1.4-6.4) | 13.6(6.0-28.5) | **<0.001** | 8.6(4.0-24.1) | 12.8(5.1-23.4) | 0.30 | 4.0(2.4-12.6) | 8.4(3.9-34.9) | **0.028** |
| **Initial treatment in hospital** | | | | | | | | | | | | |
| Anticoagulants, n (%) | 39(16.5%) | 53 (25.6%) | **0.026** | 9(34.6%) | 21(38.2%) | 0.949 | 37(36.6%) | 35(37.2%) | 1.000 | 42(70.0%) | 46(68.7%) | 1.000 |
| Antiplatelets, n (%) | 216(91.5%) | 118(57.3%) | **<0.001** | 22(84.6%) | 22(40.0%) | **<0.001** | 75(74.3%) | 65(69.1%) | 0.527 | 30(50.0%) | 26(37.8%) | 0.276 |

NOTE: HT: hemorrhagic transformation; sHT: spontaneous HT; tHT: HT after thrombectomy; TOAST: Trial of ORG 10172 in Acute Stroke Treatment; AF: atrial fibrillation; CHD: coronary heart disease; NIHSS: National Institutes of Health Stroke Scale; mRS: modified Rankin Scale; RBC: red blood cell; WBC: white blood cell; Hb: hemoglobin; PLT: platelet; ALT: alanine aminotransferase; AST: spartate amino transferase; PT: prothrombin time; APTT: activated partial thromboplastin time; TT: thrombin time; CRP: C-reaction protein.* Continuous variables were compared between the groups by the Student’s t-test or the Mann–Whitney test. The chi-square test was used for categorical variables.
